# Evaluating the frequency, prognosis and survival of RUNX1 and ASXL1 mutations in patients with acute myeloid leukaemia in northeastern Iran

**DOI:** 10.1111/jcmm.17424

**Published:** 2022-06-12

**Authors:** Mohammad Parsa‐kondelaji, Hossein Ayatollahi, Mehrdad Rostami, Maryam Sheikhi, Faezeh Barzegar, Monnavar Afzalaghaee, Elmira Moradi, Mohammad Hadi Sadeghian, Amir Abbas Momtazi‐Borojeni

**Affiliations:** ^1^ Department of Hematology and Blood Banking Faculty of Medical Sciences Mashhad University of Medical Sciences Mashhad Iran; ^2^ Department of Hematology and Blood Banking Cancer Molecular Pathology Research Center Mashhad University of Medical Sciences Mashhad Iran; ^3^ Social Determinant of Health Research Center Mashhad University of Medical Sciences Mashhad Iran; ^4^ Department of Medical Biotechnology Faculty of Medicine, Mashhad University of Medical Sciences Mashhad Iran

**Keywords:** acute myeloid leukaemia, ASXL1, mutation, prognosis, RUNX1, survival

## Abstract

To evaluate the frequency and prognosis of runt‐related transcription factor 1 (RUNX1) and additional sex combs like‐1 (ASXL1) mutations in acute myeloid leukaemia (AML) patients in northeastern Iran. This cross‐sectional study was performed on 40 patients with AML (including 35 patients with denovo AML and five patients with secondary AML) from February 2018 to February 2021. All patients were followed up for 36 months. We evaluated the frequency and survival rate of RUNX1 and ASXL1 mutations in AML patients. To detect mutations, peripheral blood samples and bone marrow aspiration were taken from all participants. One male patient (2.5%) had RUNX1 mutations and four cases (10%; 3 females vs. 1 male) had ASXL1 mutations. The survival rates of AML patients after 1, 3, 6, 9, 12, 24 and 36 months were 98%, 90%, 77%, 62%, 52%, 27% and 20%, respectively. There was a significant relationship between the occurrence of ASXL1 mutations and the survival of patients with AML (*p* = 0.027). Also, there was a significant relationship between the incidence of death and haemoglobin levels in patients with AML (*p* = 0.045). Thus, with an increase of one unit in patients' haemoglobin levels, the risk of death is reduced by 16.6%. Patients with AML had a high mortality rate, poor therapy outcome and low survival rate. ASXL1 and RUNX1 mutations are associated with a worse prognosis in patients with newly diagnosed AML. Also, we witnessed that the prevalence of ASXL1 to RUNX1 mutations was higher in northeastern Iran compared with other regions.

## 1 INTRODUCTION

Acute myeloid leukaemia (AML) has the highest mortality among the different types of leukaemias.^1^ Regarding the occurrence of AML, gene mutation plays an important role. Genomic aberrations play an important role in the pathogenesis, and cytogenetic aberrations have been recognized as well‐known diagnostic and prognostic markers.^2^, ^3^, ^4^ Currently, DNA sequencing is used to profile somatic mutations and identify recurrent somatic mutations in myeloid malignancies. These mutations along with the reported genetic changes help to determine prognostic features.^2^ Several mutated genes including runt‐related transcription factor 1 (RUNX1) and additional sex combs like‐1 (ASXL1) have been identified in AML.^2^, ^5^, ^6^ RUNX1, also known as AML1, CBFalpha2 and PEBP2alphaB,^7^ is a major transcription factor and a major regulator of haematopoiesis involved in the appearance and regulation of haematopoietic stem cells.^8^, ^9^ Differentiation of myeloid progenitor cells into granulocytes requires RUNX1, which is the most common target for chromosomal transmission associated with human leukaemia.^9^ In cytogenetically heterogeneous patients, AML mutation in RUNX1 is associated with a poor prognosis,^6^, ^8^ and the most common mutation is exon 8.^10^, ^11^ The occurrence of RUNX1 mutation with ASXL1 gene mutation has been identified in all major groups so far. ASXL1 mutations appear to be the most common simultaneous mutation with RUNX1.^9^, ^12^, ^13^ The ASXL1 gene is located in chromosome 20q11 and belongs to the gene family involved in epigenetic settings. This gene has 14 exons and is expressed in most haematopoietic cells.^14^, ^15^ Most mutations occur in exon 12 (delete from mutation type).^16^, ^17^ These genes are also involved in the silencing and expression of effective genes in haematopoiesis and leukemogenesis, respectively.^18^ ASXL1 mutations are common mutations in AML with a poor therapy outcome.^18^ Most ASXL1 mutations are nonsense and frameshift mutations, which may lead to the loss of carboxy moiety at the protein level. ASXL1 mutations frequently occur in AML patients and cause poor survival.^2^, ^19^ Accordingly, this study aimed to evaluate the frequency of RUNX1 (Exon 8) and ASXL1 (Exon 12) mutations, as the most common exon sites of mutations, and investigate the survival of AML patients with and without mutations.

## 2 MATERIALS AND METHODS

In this study, fresh bone marrow or peripheral blood samples were taken from 40 patients (20 males vs. 20 females) with newly diagnosed AML, including 35 patients with denovo AML and five patients with secondary AML (Table S1) admitted to Ghaem hospital of Mashhad, Iran, from February 2015 to February 2018. The malignancy was diagnosed and approved by one pathologist and one haematologist according to the French–American–British (FAB) criteria and the World Health Organization (WHO) standards (blood cell count, peripheral blood smear, cellular immunophenotypes and clinical symptoms).

Haematological analysis, molecular mutations analysis (Table S2) and statistical analysis are described in the supplementary material and methods.

## 3 RESULTS

### 3.1 Mutations' Frequencies

The current study enrolled 40 AML patients (20 males vs. 20 females) with a mean age of 33.22 ± 20.91 years and age range of 1–73 years. The mean age of females and males was 32.8 ± 21.9 and 33.6 ± 20.4 years, respectively. M2 and M4 were the most frequent subtypes (25%), while M0 and M6 were the least frequent subtypes (2.5%) (Table S3).

One male patient (2.5%) had RUNX1 mutations and four cases (10%; 3 females vs. 1 male) had ASXL1 mutations, with a mean age of 29.0 ± 25.80 years (Table 1). The average WBC, RBC and HB were lower and the PLT was higher in individuals with ASXL1 mutations than in individuals without the mutation. However, no significant relationship was observed between any of the laboratory indicators and the occurrence of ASXL1 mutation (*p* > 0.05). Moreover, two of the four patients with the ASXL1 mutation had the M3 subtype, while the other two had the M2 and M5 subtypes (Table 1).

**TABLE 1:** Clinical and cytogenetic characteristics according to the ASXL1 mutation status

Mutation	Sex	Age (year)	Presence of RUNX1 mutation	AML type	WBC (10^3^/μl)	RBC (10^6^/μl)	HB (g/dl)	PLT (10^3)^	Survival (month)
ASXL1	M	63	+	M5	6.2	2.84	7.8	34	1
ASXL1	F	32	−	M2	57.1	2.33	7.8	26	8
ASXL1	F	19	−	M3	80.1	2.53	8.5	95	13
ASXL1	F	2	−	M3	7.1	3.1	8.3	36	6

### 3.2 Survival analysis

**FIGURE 1:**
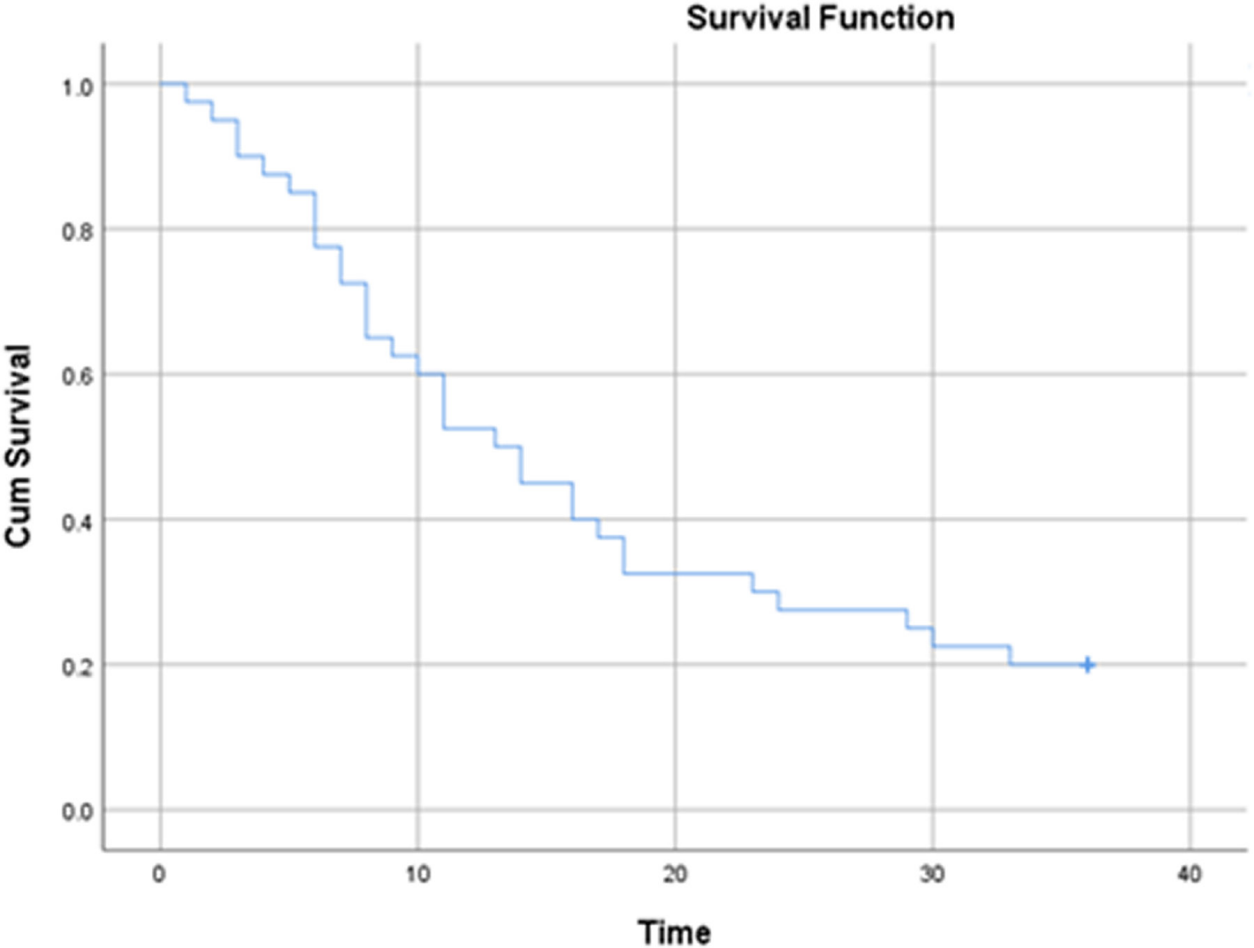
Survival of patients with acute myeloid leukaemia

**FIGURE 2:**
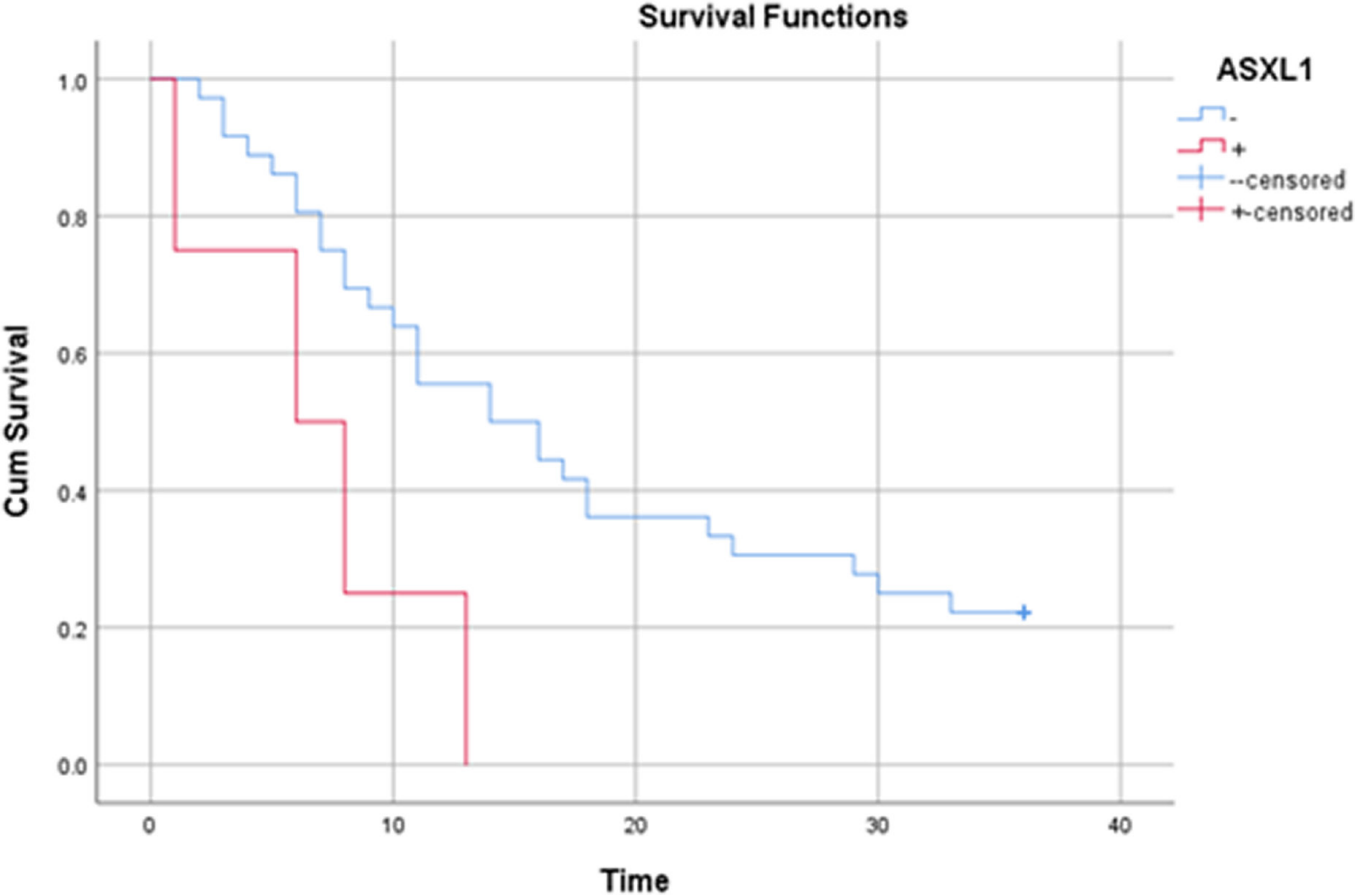
Survival of patients with acute myeloid leukaemia in terms of ASXL1 mutation

All patients were followed up for 36 months. During this period, 32 (80%) patients died. The mean duration of death was 12.2 ± 8.37 months (Table S1). The mean survival time in patients was 16.97 months (95% CI: 13.24–20.7 months) and the median survival time in patients was 13.0 months (95% CI: 8.9–17.13 months). According to the results of the life table, the survival rates of patients after 1, 3, 6, 9, 12, 24 and 36 months were 98%, 90%, 77%, 62%, 52%, 27% and 20%, respectively. Figure 1 shows the overall patient survival curve and Figure 2 presents the same figure in terms of the ASXL1 mutation.

To evaluate the relationship between patient survival and demographic variables, laboratory indices and ASXL1 mutation, a univariate Cox regression model was used (Table 2). According to the results, there was a significant relationship between the occurrence of ASXL1 mutation and the survival of patients with AML (*p* = 0.027). Accordingly, the risk of death in individuals with ASXL1 mutation was 3.48 times higher than in those who did not have a mutation. It should also be noted that all the four patients with the mutation died and their mean death time in the 36‐month follow‐up was 7.0 ± 4.9 months. In addition, there was a significant relationship between the incidence of death and haemoglobin levels in patients with AML (*p* = 0.045). Thus, with an increase of one unit in patients' haemoglobin level, the risk of death reduced by 16.6%. Also, haemoglobin levels were 7.96 ± 1.9 in those who died and 9.9 ± 2.5 in those who survived.

**TABLE 2:** Prognostic variables of survival in patients with acute myeloid leukaemia using Cox regression model

	*β*	SE	Wald	*p*‐value	HR	Lower	Upper
Sex (female)^1^	0.19	0.359	0.288	0.592	1.212	0.600	2.450
Age	−0.004	0.011	0.172	0.678	0.996	0.975	1.016
ASXL1 mutation (yes)^2^	1.25	0.563	4.904	0.027	3.482	1.154	10.503
WBC	−0.001	0.005	0.010	0.920	0.999	0.989	1.010
RBC	−0.026	0.249	0.011	0.917	0.975	0.599	1.587
HB	−0.18	0.090	4.015	0.045	0.834	0.699	0.996
PLT	0.008	0.006	1.853	0.173	1.008	0.997	1.020

*Note:* Reference level: male,^1^ No.^2^

95.0% CI for HR.

Abbreviations: CI, Confidence Interval; HR, Hazard Ratio; SE, Standard Error.

## 4 DISCUSSION

Identifying the mutations can help estimate the prognosis and survival rates and find new therapies with molecular targets. We observed a RUNX1 mutation in one (2.5%) patient with secondary AML with a previous diagnosis of CMML (Table S1). In previous studies carried out by Gaidzik et al.^11^ and Simon et al.,^20^ the frequency of RUNX1 mutations was 10%. In addition, the frequency of RUNX1 mutations in the study by You et al.^6^ was 15.1%. This discrepancy may be due to the low number of samples examined. Since this is the first study to evaluate the frequency of RUNX1 mutations in Iranian AML patients, more studies are needed in this regard. Also, the frequency of ASXL1 mutations in this study was 10%, which was consistent with the findings of previous studies. According to previous studies by Pratcorona et al.,^18^ Sasaki et al.^5^ and Fan et al.,^21^ the frequency of ASXL1 mutation in AML patients was 5.3%, 17% and 8.7%, respectively. In our study, a case of RUNX1 mutation was found, that was related to older age, male sex, presence of ASXL1 mutation and with FAB M5 morphology (Table 1); this was consistent with the general findings of Khan et al. and Gaidzik et al.^9^, ^11^ Also, in our study, due to the low prevalence of mutation RUNX1, we could not investigate the relationship between mutation and survival.

Four cases (10%) of ASXL1 mutations were observed in patients with AML, three of which were related to secondary AML (with previous diagnoses of CMML, CML and CML), and one was associated with primary AML (Table S1). Unlike the studies by Paschka et al.,^22^ Schnittger et al.,^23^ and Asada et al.^24^ in which the prevalence of ASXL1 mutations was higher in males, in our study, three of the four individuals with the ASXL1 mutation were females. The average WBC, RBC and HB were lower and the PLT was higher in individuals with ASXL1 mutations than in individuals without the mutation. However, no significant relationship was observed between any of the laboratory indicators and the occurrence of ASXL1 mutation, which confirms the findings of previous studies. The almost high prevalence of ASXL1 mutations in patients with AML, along with their very low survival, demand further studies in this field and may even lead to new AMLs being divided into groups with or without ASXL1 mutations to better predict patient survival and may change the treatment protocol.

Also, there was a significant relationship between the occurrence of ASXL1 mutation and the survival of patients with AML (*p* = 0.027), which was consistent with the study by Pratcorona et al.^18^ (*p* = 0.019). In the present study, there was a significant relationship between mortality and haemoglobin in patients with AML (*p* = 0.045). Therefore, low haemoglobin can be considered as a sign of severe acute illness and it can reduce the chance of treatment; previous studies confirm the role of haemoglobin in predicting the survival rate of AML patients.^25^ ASXL1 mutation is common in patients with AML, and it has a low survival rate and high mortality. Thus, blocking ASXL1 activity might greatly enhance the current therapeutic measurements. However, considering the prevalence and adverse outcome of ASXL1 mutations in AML, it is essential to identify the molecular landscape of ASXL1+ AML patients for establishing precise risk stratification in this subgroup of AML.

Since these two genes are involved in haematopoiesis and leukogenesis, it is recommended that further studies be conducted to evaluate them. According to the results, the identification of mutations can be used as a marker with diagnostic value and appropriate treatment. However, further studies with larger sample size are recommended to confirm our results.

## AUTHOR CONTRIBUTIONS

**Mohammad Parsa‐kondelaji:** Investigation (lead); writing – original draft (lead). **Hossein Ayatollahi:** Methodology (equal); writing – original draft (supporting). **Mehrdad Rostami:** Formal analysis (lead). **Maryam Sheikhi:** Writing – review and editing (lead). **Faezeh Barzegar:** Investigation (supporting). **Monnavar Afzalaghaee:** Methodology (equal). **Elmira Moradi:** Methodology (equal). **Mohammad Hadi Sadeghian:** Conceptualization (lead); funding acquisition (lead). **Amir Abaas Momtazi‐Borojeni:** Supervision (equal); validation (equal); writing – review and editing (lead).

## CONFLICT OF INTEREST

The authors declare that there are no conflicts of interest.

## CODE AVAILABILITY

Not applicable.

## Supporting information


Appendix S1

## Data Availability

The data that support the findings of this study are available from the corresponding author upon reasonable request.
