# Supplementary material for: Evaluating the frequency, prognosis and survival of RUNX1 and ASXL1 mutations in patients with acute myeloid leukaemia in northeastern Iran
Source: J Cell Mol Med. 2022 Jun 12;26(13):3797–801. doi: 10.1111/jcmm.17424 (PMC9258702; doi:10.1111/jcmm.17424)
Supplement: Supplementary file 1 — Appendix S1 [file JCMM-26-3797-s001.docx]

**Supplementary Materials and methods**

***Hematological Analysis***

Samples were analyzed utilizing an SYSMEX automated hematology analyzer. The following parameters were measured for all samples: White Blood Cells (WBC), Red Blood Cells (RBC), Platelet count (PLT), Hemoglobin (Hb), and Hematocrit (Hct).

***Molecular Mutations Analysis***

DNA was extracted from 40 bone marrow aspiration and peripheral blood using the Gene bio kit according to the DNA isolation instructions written on it for mutation detecting. Polymerase chain reaction (PCR) was performed by the ABI machine. For the RUNX1 (Exon 8), the sequence of forward primer was F: 5’-CGCAACCTCCTACTCACTTC- 3' and reverse primer was R: 5’-TGACCTACAGCGAGATCCTG-3' (Table S2). The cycling program of PCR for amplification of RUNX1 mutation was holding at 94°C for 5 min, 30 s at 94°C, 30 s at 61°C, 30 s at 60°C (the last three steps were repeated 40 cycles), and 5 min at 72°C. The PCR reaction was conducted over 35 cycles for 1 min at 4°C, 30 s at 60°C, and 90 s at 72°C. Also, the forward primer used for ASXL1 (Exon12) mutation was 5’-TGCCATGACCCTTAAGCTACT-3' and reverse primer was 5'-AAGGCGGCAGTAGTTGTGTT-3' (Table S2). All amplified DNAs were loaded on the 2% agarose (Ladder: YTA 50 bp DNA) electrophoresis gel. The agarose gel electrophoresis voltage system was set on 90-100 volt. After 15-20 min, the gel was ready for examination under the ultraviolet (UV) light. We reached a single and clean DNA banding on the agarose gel. After ensuring amplified DNA, the samples were sent for sequencing and the results were analyzed by CLC software.

***Statistical Analysis***

In this study, we investigated the relationship between gene mutations and other variables. Fisher’s exact test was used to compare the differences between categorical variables. Mann-Whitney U test was applied to compare the differences in continuous variables according to mutations. Furthermore, the associated factors with death due to cancer were determined using univariate Cox models. All data analyses were performed using SPSS version 16.0 and *P*<0.05 was considered a statistically significant difference.

| **Table S1.** Characteristics of de novo AML and secondary AML. | | | | | | | | | | | |
| --- | --- | --- | --- | --- | --- | --- | --- | --- | --- | --- | --- |
| Diagnosis | Survival  (Month) | PLT  [10^3^} | HB  [g/dl] | RBC  [10^6^/μL] | WBC  [10^3^/μL] | AML | ASXL1 | RUNX1 | SEX | AGE  (Year) | Type of AML |
| Cytomorphology, cytochemistry, and flow cytometry | 9 | 36 | 11.4 | 3.43 | 9.8 | M4 | Neg | Neg | F | 35 | Denovo |
|  | 8 | 13 | 8.1 | 2.74 | 45.7 | M1 | Neg | Neg | F | 62 |  |
|  | Live | 58 | 13.9 | 3.74 | 47.7 | M4 | Neg | Neg | F | 32 |  |
|  | 16 | 26 | 8.1 | 61.9 | 61.9 | M4 | Neg | Neg | F | 5 |  |
|  | 13 | 95 | 8.5 | 2.53 | 80.1 | M3 | Pos | Neg | F | 19 |  |
|  | Live | 71 | 11.3 | 3.56 | 104.9 | M4 | Neg | Neg | F | 31 |  |
|  | 11 | 40 | 7.6 | 2.9 | 93.4 | M2 | Neg | Neg | F | 22 |  |
|  | 6 | 108 | 6.1 | 3.7 | 96.3 | M1 | Neg | Neg | F | 4 |  |
|  | 7 | 99 | 6.1 | 1.89 | 95.2 | M2 | Neg | Neg | F | 14 |  |
|  | 16 | 110 | 8.4 | 2.78 | 99.7 | M3 | Neg | Neg | F | 55 |  |
|  | 18 | 79 | 7.1 | 2.2 | 58.2 | M5 | Neg | Neg | F | 60 |  |
|  | 18 | 68 | 8.3 | 1.89 | 92.9 | M4 | Neg | Neg | F | 17 |  |
|  | 17 | 104 | 6.8 | 2.8 | 71.7 | M2 | Neg | Neg | F | 68 |  |
|  | 14 | 19 | 10.9 | 3.83 | 43.5 | M2 | Neg | Neg | F | 66 |  |
|  | Live | 27 | 7.1 | 2.44 | 39.4 | M4 | Neg | Neg | F | 36 |  |
|  | 8 | 30 | 8.7 | 2.72 | 29.6 | M3 | Neg | Neg | F | 7 |  |
|  | 4 | 27 | 8.1 | 2.67 | 87.5 | M5 | Neg | Neg | M | 5 |  |
|  | 30 | 54 | 7.8 | 2.28 | 71.4 | M2 | Neg | Neg | M | 35 |  |
|  | 3 | 108 | 7.4 | 1.69 | 99.7 | M4 | Neg | Neg | M | 49 |  |
|  | 24 | 36 | 5.5 | 1.76 | 5.5 | M5 | Neg | Neg | M | 60 |  |
|  | 11 | 52 | 4.9 | 1.67 | 6.4 | M2 | Neg | Neg | M | 21 |  |
|  | 23 | 60 | 9 | 2.56 | 99.8 | M2 | Neg | Neg | M | 41 |  |
|  | Live | 56 | 7.3 | 1.86 | 99.9 | M1 | Neg | Neg | M | 38 |  |
|  | 3 | 186 | 12.2 | 5.09 | 26.7 | M4 | Neg | Neg | M | 1 |  |
|  | Live | 11 | 7.4 | 2.28 | 14.4 | M6 | Neg | Neg | M | 33 |  |
|  | 14 | 105 | 8.7 | 2.62 | 96.9 | M0 | Neg | Neg | M | 44 |  |
|  | 10 | 67 | 6.3 | 1.82 | 83.1 | M4 | Neg | Neg | M | 73 |  |
|  | 11 | 28 | 10.7 | 3.64 | 40.3 | M1 | Neg | Neg | M | 46 |  |
|  | 7 | 31 | 7.9 | 3.61 | 26.3 | M2 | Neg | Neg | M | 4 |  |
|  | Live | 79 | 10.8 | 3.28 | 80.8 | M3 | Neg | Neg | M | 19 |  |
|  | 6 | 19 | 2.08 | 2.08 | 38.8 | M5 | Neg | Neg | M | 9 |  |
|  | 33 | 54 | 8.2 | 1.9 | 22.5 | M4 | Neg | Neg | M | 48 |  |
|  | 2 | 65 | 7.2 | 2.08 | 57.3 | M3 | Neg | Neg | M | 33 |  |
|  | Live | 100 | 9.1 | 2.91 | 52.7 | M1 | Neg | Neg | M | 16 |  |
|  | Live | 39 | 12.3 | 4.13 | 18.2 | M3 | Neg | Neg | M | 34 |  |
| CML | 5 | 93 | 10.1 | 4.27 | 71.2 | M1 | Neg | Neg | F | 56 | Secondary |
| CML | 6 | 36 | 8.3 | 3.1 | 7.1 | M3 | Pos | Neg | F | 2 |  |
| MDS | 29 | 39 | 8.7 | 3.14 | 20.4 | M2 | Neg | Neg | F | 34 |  |
| CML | 8 | 26 | 7.8 | 2.35 | 57.1 | M2 | Pos | Neg | F | 32 |  |
| CMML | 1 | 134 | 7.8 | 2.84 | 6.2 | M5 | Pos | Pos | M | 63 |  |

**Table S2.** Sequences of primers for PCR

| **Size** | **Reverse Primer** | **Forward Primer** |  |
| --- | --- | --- | --- |
| 683kb | 5’- TGACCTACAGCGAGATCCTG-3’ | 5’-CGCAACCTCCTACTCACTTC-3’ | **RUNX1**  **(Exon 8)** |
| 479kb | 5’- AAGGCGGCAGTAGTTGTGTT-3’ | 5’- TGCCATGACCCTTAAGCTACT-3’ | **ASXL1**  **(Exon 12)** |

**Table S3.** Distribution of demographic and laboratory indicators in patients with acute myeloid leukemia based on ASXL1 mutations.

| Variable | All patients | ASXL1 mutations | | *P*-value |
| --- | --- | --- | --- | --- |
|  |  | Yes | No |  |
| Age (years) | 33/23 ± 20/91  33/50 (1-73) | 29/00 ± 25/80  25/50 (2-63) | 33/69 ± 20/69  34/00 (1-73) | 0/586 |
| Sex (male/ female) | 20/20 | 3/1 | 17/19 | 0/605 |
| M0  M1  M2  M3  M4  M5  M6 | (% 2/5) 1  (% 15/0) 6  (%25/0) 10  (% 17/5) 7  (% 25/0) 10  (% 12/5) 5  (% 2/5) 1 | 0  0  (% 10/0) 1  (% 28/6) 2  0  (% 20/0) 1  0 | (%100) 1  (% 100) 6  (% 90/0) 9  (% 71/4) 5  (%100) 10  (% 80/0) 4  (% 100) 1 | 0/439 |
| WBC | 56/50 ± 33/0  (5/5 – 104/9) 57/2 | 37/62 ± 36/98  (6/2 – 80/10) 32/1 | 58/60 ± 32/42  (5/5 – 104/9) 57/7 | 0/207 |
| RBC | 2/79 ± 0/8  (1/67 – 5/09) 2/73 | 2/70 ± 0/33  (2/35 – 3/10) 2/68 | 2/80 ± 0/84  (1/67 – 5/09) 2/73 | 0/983 |
| HB | 8/35 ± 2/18  (2/08 – 13/9) 8/1 | 8/10 ± 0/35  (7/8 – 8/5) 8/05 | 8/37 ± 2/30  (2/08 – 13/9) 8/1 | 0/983 |
| PLT | 62/20 ± 38/24  (11 – 186) 55 | 72/75 ± 50/93  (26 – 134) 65/5 | 61/03 ± 37/32  (11 – 186) 55 | 0/778 |
